# Establishment of a simple method to evaluate mixing times in a plastic bag photobioreactor using image processing based on freeware tools

**DOI:** 10.1186/s13104-021-05892-2

**Published:** 2021-12-29

**Authors:** Henrike Wurm, Michael Sandmann

**Affiliations:** University of Applied Sciences Neubrandenburg, Brodaer Straße 2, 17033 Neubrandenburg, Germany

**Keywords:** Mixing time, Image analysis, Photobioreactor, pH probe, Aeration, Algae, Fluid dynamics

## Abstract

**Objective:**

Accurate determination of the mixing time in bioreactors is essential for the optimization of the productivity of bioprocesses. The aim of this work was to develop a simple optical method to determine the mixing time in a photobioreactor. The image processing method should be based on freeware tools, should not require programming skills, and thus could be used in education within high schools and in early stages of undergraduate programs.

**Results:**

An optical method has been established to analyze images from recorded videos of mixing experiments. The steps are: 1. Extraction of a sequence of images from the video file; 2. Cropping of the pictures; 3. Background removal; and 4. Image analysis and mixing time evaluation based on quantification of pixel-to-pixel heterogeneity within a given area of interest. The novel method was generally able to track the dependency between aeration rate and mixing time within the investigated photobioreactor. In direct comparison, a pearson correlation coefficient of rho = 0.99 was obtained. Gas flow rates between 10 L h^−1^, and 300 L h^−1^ resulted from mixing times of between 48 and 14 s, respectively. This technique is applicable without programming skills and can be used in education with inexperienced user groups.

## Introduction

Microalgae have attracted much attention due to their wide range of possible uses [1–7]. Algal cells are often grown under controlled conditions in closed photobioreactors (PBR). PBR provide better control of contamination and cell physiology than open systems, resulting in better growth and better quality of the harvested product. The major disadvantages for PBR are high initial investment costs and operating costs [8, 9]. In recent years, plastic bag PBR for commercial production of microalgae have attracted considerable attention due to their low investment costs [10–14]. On the other hand, plastic bag PBR often have a reduced lifespan and inherent fragility, and they are known to suffer from inadequate mixing and decreased cell growth [15]. Proper mixing in reactors is needed, e.g., for optimal mass and heat transfer and to prevent cells settling. Inadequate mixing is the reason for the appearance of spatial gradients in reactors, which is believed to inherently increase cell-to-cell heterogeneity and thus negatively affect the productivity of bioprocesses and product quality [2, 16–18].

Mixing time is defined as the time needed to obtain prescribed uniformity of a tracer (usually a pulse input) in a reactor that is close to a complete mixed state. In practice, a state of homogeneity with ± 5% deviation is often the target [19]. Because of the considerable importance of this topic in industrial processes, several methods have been developed to measure mixing times, including conductivity and pH tracer experiments or colorimetric measurements [20, 21]. Digital image analysis has been increasingly recognized as a valuable tool for analyzing mixing behavior in complex reactor geometries. These techniques eliminate subjective estimates of mixing times by the human eye. One clear disadvantage is that programming skills are often needed to run the analysis automatically, thus limiting its applicability for a broader user group. In the field of PBR, “classical” mixing time analyses are rare. To the best of our knowledge, image-based mixing time analyses have not been applied in PBR before. To overcome this barrier, a simple method based on image processing using free software tools has been established to characterize mixing behavior in a self-constructed plastic bag PBR. A “classical” pH tracer experiment was used as reference analysis [22]. A very high correlation between both methods was obtained illustrating the suitability of the novel method.

## Main text

### Experimental section

#### Plastic bag photo bioreactor

**Fig. 1:**
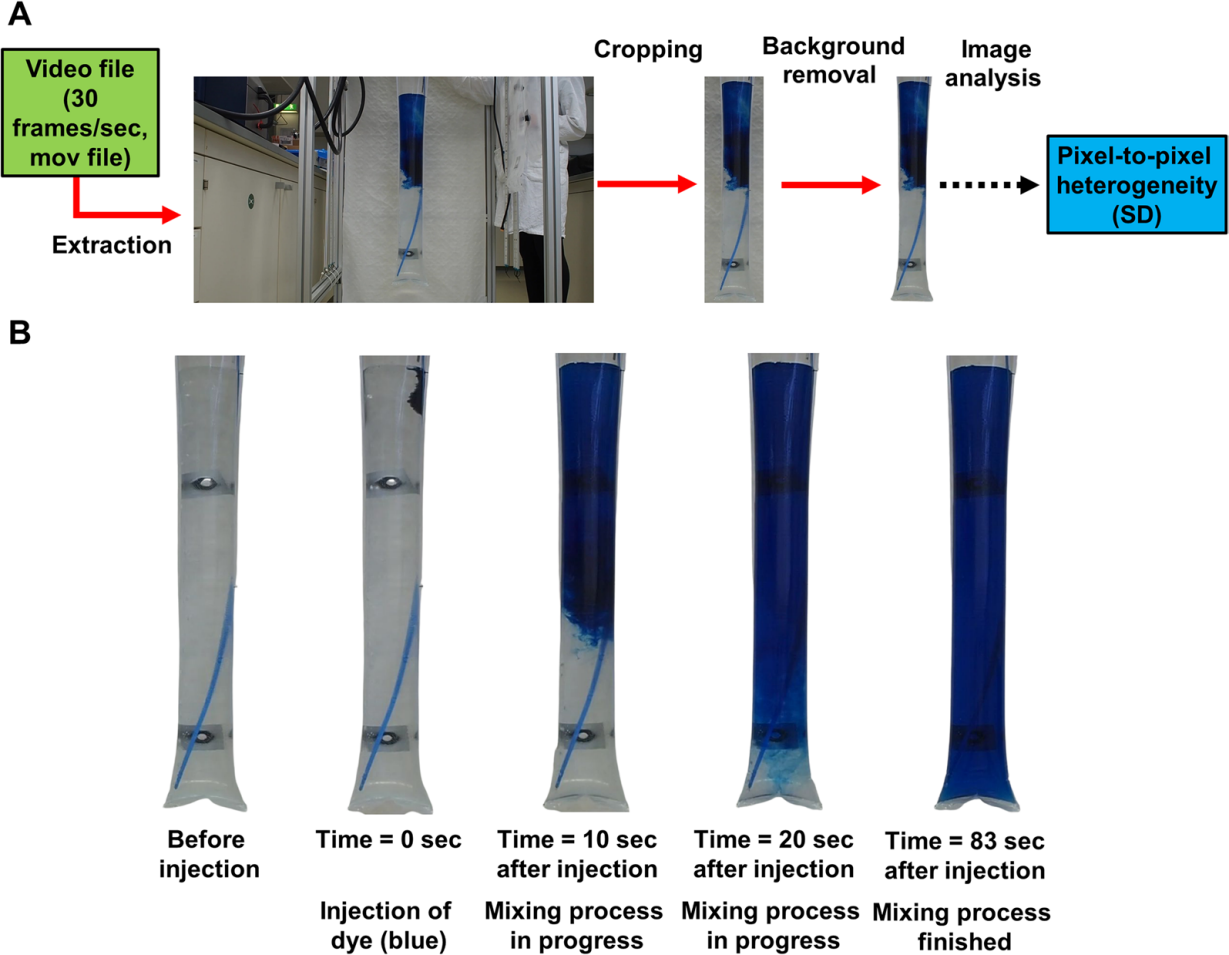
Experimental details. **A** Image processing flow chart. **B** Example of frames extracted from a sequence of images captured during a mixing process (gas flow rate 10 L h^−1^)

Analysis was performed in a self-constructed plastic bag PBR which operates on the bubble column principle (Fig. 1). Briefly, the PBR has a total height of 1.9 m, a width of 0.8 m and consists of a metal frame made of aluminum sections (MiniTec, Schönenberg-Kübelberg, Germany). Up to three transparent plastic bags (11 cm diameter) can be mounted on top of the frame. The bags used (CASO Design Braukmann, Arnsberg, Germany) consist of an outer polyamide layer and an inner polyethylene layer that are 15 µm and 135 µm thick, respectively. The original purpose of these bags has been vacuum-packing of food which implies an inherent food safety that will be beneficial for algal cultivation. The bags are closed on the bottom with a foil heat sealer and can be filled with different volumes of water or culture media for algal growth. Six 1.16 m long dimmable light-emitting diode modules (LEDaquaristik, Hövelhof, Germany) illuminate the algal cells. The bags were aerated with 6 mm tubes made of nylon (RS Components, Frankfurt, Germany). The aeration tubes were inserted from the top of the reaction vessel and then guided to the bottom. The bags were additionally prepared with cord grips (16 mm diameter) that act as ports for sampling or integration of measuring probes, for example. The mixing time experiments were performed at room temperature (23 °C) with 10 L of distilled water and with different gas flow rates (10 L h^−1^, 50 L h^−1^, 100 L h^−1^, 300 L h^−1^). Mixing was done with nitrogen. Statistical analyses (arithmetic mean, standard deviations and correlation analysis) have been done with SigmaPlot® (Systat Software GmbH, Düsseldorf, Germany).

#### Reference method (pH probe)

The reference method was adapted after Zhang et al. [22]. Sodium hydroxide was used as a tracer (5 mL of 38 mM NaOH stock solution). The pH probe (EasyFerm Bio HB Arc 120 by Hamilton Bonadaz AG) was immersed 30 cm below the surface of the liquid (upper third of the liquid level) and tracked the changes in pH over time with a measurement interval of 3 s. A manual syringe with a small tube was used to apply the solution directly to the surface of the fluid. The injection duration was a fraction of second. After the experiment was finished, the bubble column was emptied with a peristaltic pump, rinsed with distilled water, and refilled for the next experiment. Mixing time was noted as the time from addition of the tracer until ± 5% of its final stable value (defined as the mean of the last 15 values) was reached. The reference experiments were performed in duplicate.

#### Optical dye tracing experiments

For these experiments, mixing of a dye was observed with a video camera and subsequently analyzed. The digital camera (Olympus OM-D E-M10 Mark II, Olympus Europa, Hamburg, Germany) was placed in front of the bubble column (mov format, 30 frames/sec). A white background was also placed behind the bubble column to minimize disturbing background signals. The blue food dye (patent blue, E131) (Brauns Heitmann, Warburg, Germany) was added with a manual syringe directly to the surface of the fluid (0.075 g powder was pre-mixed with 5 mL of distilled water). The injection duration was a fraction of second. The bubble columns were emptied after each mixing experiment with a peristaltic pump, rinsed with distilled water, and refilled for the next experiment.

#### Image analysis based on freeware

The key steps of the image preparation were as follows (Fig. 1):

1. Extraction and storage of a sequence of images from the original video file (sampled at 30 images/sec) was done automatically at a frequency of one image/sec with the tool VLC media player 3.0.16 (VideoLAN, Paris, France).
2. Cropping of the picture was performed automatically with IrfanView 4.58 so that only the bubble columns and a reduced background area are visible.
3. Final background removal was performed with the on-line tool removebg (Kaleido AI, Wien, Austria). After automated processing, the files were again stored in the computer’s memory and further analyzed.
4. Image analysis and mixing time evaluation rely on quantification of pixel-to-pixel heterogeneity within a given area of interest, which can be performed using the standard deviation (SD) across all pixels used. With this method, the whole image of the bubble column was used after background removal. Standard deviations were determined using Gimp 2.10.24 (GNU Image Manipulation Program). To evaluate the pixel-to-pixel heterogeneity based on the pixel intensities, the GIMP feature “value” was used. This “pseudochannel” simulates a greyscale image. Thus the color information is reduced without an additional work step.

### Results

**Fig. 2:**
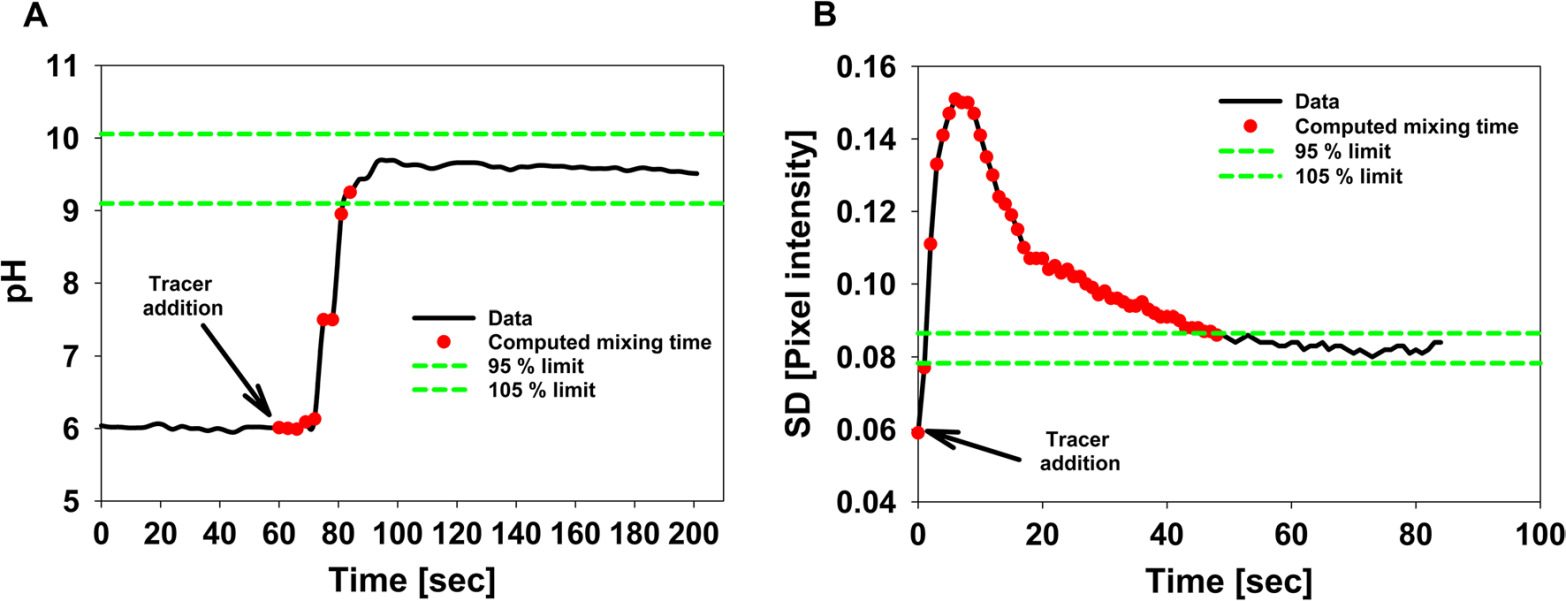
Determination of mixing times in the 10 L bubble column at a gas flow rate of 10 L h^−1^. The mixing times have been evaluated between tracer additions until signal stability of ± 5.0% was reached (evaluated length of mixing time marked with red dots). **A** Reference method (pH probe). **B** Optical dye tracing method

The pH tracer method as well as the novel image processing based method for determining the mixing times within a self-constructed 10 L bubble column PBR relied on typical tracer experiments. Exemplary data sets are shown in Fig. 2. Compared to the classical pH tracer method, a novel method that is based on the use of freeware tools for image analysis was established. In both methods, the time dependent development of a signal (pH in solution or pixel-to-pixel heterogeneity within a given image) was used to determine mixing times. The measurement intervals for the pH method and the optical analysis were 3 s and 1 s, respectively. Pixel-to-pixel heterogeneity, as measured by the SD, exhibits minimal values before addition of the tracer and after complete mixing. During the mixing process, SD is dynamic and relatively high.

**Fig. 3:**
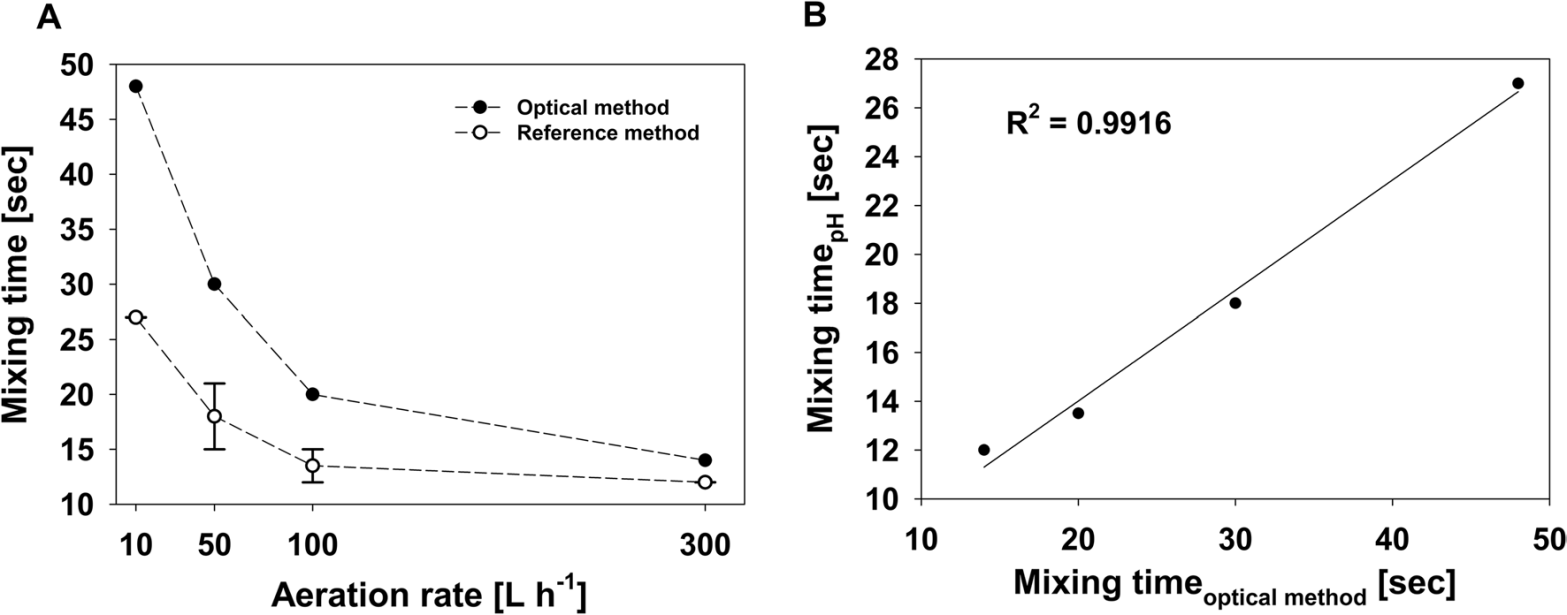
Comparison of methods. **A** Effect of different aeration rates on mixing time (mean and standard deviation are shown for reference analysis, n = 2). **B** Correlation of mixing times derived from optical dye tracing method with reference analysis

It is seen in Fig. 3A that the mixing time decreases as the aeration rate increases but the results obtained with the optical method are always higher. The mixing times that were measured range between 48 and 14 s for the optical method and between 27 and 12 s for the reference method (Fig. 3A). In a direct comparison of all mixing times obtained, a relatively high linear correlation was found (R^2^ = 0.9916).

### Discussion

In this work a simple method based on image processing using free software tools has been established to evaluate mixing times in PBR. The technique was applied to a self-constructed plastic bag PBR and was compared to an established reference analysis. The optical method uses free software packages that can be applied without any programming skills and is based on characterization of pixel-to-pixel heterogeneity within a sequence of images of the mixing process within the reactor (Fig. 1). The established simple method uses the pixel-to-pixel heterogeneity, which is represented by the SD over all pixel intensities in a given area of interest. During the mixing process, the SD is very dynamic and high because the dye that is used is only partly distributed in the bubble column. As the mixed state is reached, the SD decreases and reaches a very low value after complete mixing (Fig. 2). The optical method consistently resulted in higher mixing times compared to the reference technique (Fig. 3) but was able to track the general relationship between aeration rate and mixing time as determined by the reference method. Both techniques showed a high linear correlation (R^2^ = 0.9916). The systematically higher mixing times can be explained by two factors. First, the relatively short distance between the injection point of the tracer solution and the insertion location of pH probe. This might result in a reduced mixing time, even though complete mixing is not guaranteed. Second, the images obtained clearly show that the bottom of the bubble column is mixed in the last phase (Fig. 2). Thus, the image-based technique can inherently identify segregated regions or dead zones and yields more reliable macromixing information. Another advantage of the optical method is the high time resolution, which can be as high as the intrinsic frame rate of the video. Compared to the mixing times of other PBR, the values obtained (between 48 and 14 s for the optical method) are relatively low, indicating good mixing behavior. In a rectangular airlift loop PBR, mixing times of 30 to 200 s have been described [23]. Another loop PBR ranged between 20 und 160 s [24]. Even higher mixing times of 5 to 15 min are possible in tubular and flat panel PBR [25, 26]. Despite these more commonly used types of PBR much more complex geometries have been developed to improve their productivity. One example is the so-called “mesh ultra-thin layer PBR” (MUTL-PBR) in which the algal suspension is distributed within an airspace containing a 3D-matrix [10, 27]. The matrix decelerates the movement of the cells within in the airspace, putatively facilitating a better mass transfer and light penetration. In this type of reactor, the cells are not equally distributed, and a significant proportion of cells is even immobilized on the 3D-matrix [27]. In such complex reactor geometry, the analysis of mixing times would likely result in very high mixing times because of the complex interaction between the 3D-matrix, the liquid phase, and the gas phase. On the other side, such a putatively high mixing time does not necessarily influence the majority of the cells, because mixing inside a “core region” of the MUTL-PBR might be very effective. The superior performance of this reactor concept has been proven in trials [10, 27, 28]. This indicates that the mixing time is important but is not the only parameter limiting PBR performance. In terms of PBR performance at least the light penetration should be also considered [29].

It can be concluded that in relation to other image-based methods [21, 30, 31], the described optical method can be implemented without commercial software and programming skills. Additionally, no advanced mathematical data handling steps are needed, such as discrimination between mixed and unmixed pixel populations [30]. Thus, the methodology can be used in education within high schools and in early stages of undergraduate programs to demonstrate the importance of mixing times in reactors. Additionally, it can be used as didactic tool to illustrate the potential workflow in image processing routines before acquiring programming skills. Despite the simplicity of the image-based method, it can be applied only in transparent reactors like PBR.

### Limitations

No replicate experiments were performed for the evaluation of the mixing times with the optical method. Nevertheless, the data acquired seem to be strongly comparable to the reference analysis.

## Data Availability

The original datasets and other miscellaneous materials related to the investigation are available upon reasonable request to the corresponding author.

## Abbreviations

sec: Second
PBR: Photobioreactor
SD: Standard deviation
Fig: Figure
MUTL: Mesh ultra-thin layer

## References

[1] de Jaeger L, Verbeek RE, Draaisma RB, Martens DE, Springer J, Eggink G, Wijffels RH (2014). Superior triacylglycerol (TAG) accumulation in starchless mutants of *Scenedesmus obliquus*: (I) mutant generation and characterization. Biotechnol Biofuels.

[2] Sandmann M, Schafberg M, Lippold M, Rohn S (2018). Analysis of population structures of the microalga *Acutodesmus obliquus* during lipid production using multi-dimensional single-cell analysis. Sci Rep.

[3] Wells ML, Potin P, Craigie JS, Raven JA, Merchant SS, Helliwell KE, Smith AG, Camire ME, Brawley SH (2017). Algae as nutritional and functional food sources: revisiting our understanding. J Appl Phycol.

[4] Zhu L (2015). Biorefinery as a promising approach to promote microalgae industry: An innovative framework. Renew Sust Energ Rev.

[5] Smetana S, Sandmann M, Rohn S, Pleissner D, Heinz V (2017). Autotrophic and heterotrophic microalgae and cyanobacteria cultivation for food and feed: Life Cycle Assessment. Bioresour Technol.

[6] Sevgili H, Sezen S, Yılayaz A, Aktaş Ö, Pak F, Aasen IM, Reitan KI, Sandmann M, Rohn S, Turan G, Kanyılmaz M (2019). Apparent nutrient and fatty acid digestibilities of microbial raw materials for rainbow trout (Oncorhynchus mykiss) with comparison to conventional ingredients. Algal Res.

[7] Hensel B, Jakop U, Scheinpflug K, Schröter F, Sandmann M, Mühldorfer K, Schulze M (2021). Low temperature preservation: influence of putative bioactive microalgae and hop extracts on sperm quality and bacterial load in porcine semen. Sustain Chem Pharm..

[8] Gupta PL, Lee SM, Choi HJ (2015). A mini review: photobioreactors for large scale algal cultivation. World J Microbiol Biotechnol.

[9] Gifuni I, Pollio A, Safi C, Marzocchella A, Olivieri G (2018). Current bottlenecks and challenges of the microalgal biorefinery. Trends Biotechnol.

[10] Schreiber C, Behrendt D, Huber G, Pfaff C, Widzgowski J, Ackermann B, Müller A, Zachleder V, Moudříková Š, Mojzeš P, Schurr U, Grobbelaar J, Nedbal L (2017). Growth of algal biomass in laboratory and in large-scale algal photobioreactors in the temperate climate of western Germany. Bioresour Technol.

[11] Wang B, Lan CQ, Horsman M (2012). Closed photobioreactors for production of microalgal biomasses. Biotechnol Adv.

[12] Shastik E, Romanova A, Laurinavichene T, Petushkova E, Sakurai H, Tsygankov A (2020). Plastic bags as simple photobioreactors for cyanobacterial hydrogen production outdoors in Moscow region. Int J Energy Environ Eng.

[13] Romero-Villegas GI, Fiamengo M, Acién Fernández FG, Molina GE (2018). Utilization of centrate for the outdoor production of marine microalgae at pilot-scale in flat-panel photobioreactors. J Biotechnol.

[14] Tredici MR, Bassi N, Prussi M, Biondi N, Rodolfi L, Chini Zittelli G, Sampietro G (2015). Energy balance of algal biomass production in a 1-ha “Green Wall Panel” plant: How to produce algal biomass in a closed reactor achieving a high Net Energy Ratio. Appl Energy.

[15] Huang Q, Jiang F, Wang L, Yang C (2017). Design of photobioreactors for mass cultivation of photosynthetic organisms. Engineering.

[16] Enfors SO, Jahic M, Rozkov A, Xu B, Hecker M, Jürgen B, Krüger E, Schweder T, Hamer G, O’Beirne D, Noisommit-Rizzi N, Reuss M, Boone L, Hewitt C, McFarlane C, Nienow A, Kovacs T, Trägårdh C, Fuchs L, Revstedt J, Friberg PC, Hjertager B, Blomsten G, Skogman H, Hjort S, Hoeks F, Lin HY, Neubauer P, van der Lans R, Luyben K, Vrabel P, Manelius Å (2001). Physiological responses to mixing in large scale bioreactors. J Biotechnol.

[17] Lee JA, Riazi S, Nemati S, Bazurto JV, Vasdekis AE, Ridenhour BJ, Remien CH, Marx CJ (2019). Microbial phenotypic heterogeneity in response to a metabolic toxin: Continuous, dynamically shifting distribution of formaldehyde tolerance in Methylobacterium extorquens populations. PLoS Genet.

[18] Dusny C, Grünberger A (2020). Microfluidic single-cell analysis in biotechnology: from monitoring towards understanding. Curr Opin Biotechnol.

[19] Nienow AW (1997). On impeller circulation and mixing effectiveness in the turbulent flow regime. Chem Eng Sci.

[20] Rosseburg A, Fitschen J, Wutz J, Wucherpfennig T, Schlüter M (2018). Hydrodynamic inhomogeneities in large scale stirred tanks—Influence on mixing time. Chem Eng Sci.

[21] Cabaret F, Bonnot S, Fradette L, Tanguy PA (2007). Mixing time analysis using colorimetric methods and image processing. Ind Eng Chem Res.

[22] Zhang A, Tsang VL, Korke-Kshirsagar R, Ryll T (2014). Effects of pH probe lag on bioreactor mixing time estimation. Process Biochem.

[23] Guo X, Yao L, Huang Q (2015). Aeration and mass transfer optimization in a rectangular airlift loop photobioreactor for the production of microalgae. Bioresour Technol.

[24] Pirouzi A, Nosrati M, Shojaosadati SA, Shakhesi S (2014). Improvement of mixing time, mass transfer, and power consumption in an external loop airlift photobioreactor for microalgae cultures. Biochem Eng J.

[25] Oncel S, Kose A (2014). Comparison of tubular and panel type photobioreactors for biohydrogen production utilizing Chlamydomonas reinhardtii considering mixing time and light intensity. Bioresour Technol.

[26] Ugwu CU, Aoyagi H (2011). Evaluation of the mass transfer capacity of a long tubular photobioreactor with static mixer and its outdoor performance with microalgal cultures. Trends Appl Sci Res.

[27] Sandmann M, Smetana S, Heinz V, Rohn S (2021). Comparative life cycle assessment of a mesh ultra-thin layer photobioreactor and a tubular glass photobioreactor for the production of bioactive algae extracts. Bioresour Technol.

[28] Pulz O, Broneske J, Waldeck P. IGV GmbH Experience Report, Industrial Production of Microalgae Under Controlled Conditions: Innovative Prospects. In: Handbook of Microalgal Culture: Applied Phycology and Biotechnology. Chichester: Wiley; 2013. p. 445–460.

[29] Laifa R, Morchain J, Barna L, Guiraud P (2021). A numerical framework to predict the performances of a tubular photobioreactor from operating and sunlight conditions. Algal Res.

[30] Vega-Alvarado L, Taboada B, Hidalgo-Millán A, Ascanio G (2011). An image analysis method for the measurement of mixing times in stirred vessels. Chem Eng Technol.

[31] Melton L, Lipp C, Spradling R, Paulson KA (2002). Dismt - Determination of mixing time through color changes. Chem Eng Commun.

